# The Impact of Text Message Reminders on Adherence to Antimalarial Treatment in Northern Ghana: A Randomized Trial

**DOI:** 10.1371/journal.pone.0109032

**Published:** 2014-10-28

**Authors:** Julia R. G. Raifman, Heather E. Lanthorn, Slawa Rokicki, Günther Fink

**Affiliations:** 1 Department of Global Health and Population, Harvard School of Public Health, Boston, MA, United States of America; 2 Harvard School of Public Health, Boston, MA, United States of America; 3 Department of Health Policy, Harvard Graduate School of Arts and Sciences, Cambridge, MA, United States of America; Division of Parasitic Diseases and Malaria, Center for Global Health, United States of America

## Abstract

**Background:**

Low rates of adherence to artemisinin-based combination therapy (ACT) regimens increase the risk of treatment failure and may lead to drug resistance, threatening the sustainability of current anti-malarial efforts. We assessed the impact of text message reminders on adherence to ACT regimens.

**Methods:**

Health workers at hospitals, clinics, pharmacies, and other stationary ACT distributors in Tamale, Ghana provided flyers advertising free mobile health information to individuals receiving malaria treatment. The messaging system automatically randomized self-enrolled individuals to the control group or the treatment group with equal probability; those in the treatment group were further randomly assigned to receive a simple text message reminder or the simple reminder plus an additional statement about adherence in 12-hour intervals. The main outcome was self-reported adherence based on follow-up interviews occurring three days after treatment initiation. We estimated the impact of the messages on treatment completion using logistic regression.

**Results:**

1140 individuals enrolled in both the study and the text reminder system. Among individuals in the control group, 61.5% took the full course of treatment. The simple text message reminders increased the odds of adherence (adjusted OR 1.45, 95% CI [1.03 to 2.04], p-value 0.028). Receiving an additional message did not result in a significant change in adherence (adjusted OR 0.77, 95% CI [0.50 to 1.20], p-value 0.252).

**Conclusion:**

The results of this study suggest that a simple text message reminder can increase adherence to antimalarial treatment and that additional information included in messages does not have a significant impact on completion of ACT treatment. Further research is needed to develop the most effective text message content and frequency.

**Trial Registration:**

ClinicalTrials.gov NCT01722734

## Introduction

Despite massive international efforts over the past decades, malaria continues to be one of the primary causes of mortality worldwide. An estimated 655,000 to 1.24 million people died of malaria in 2010, and more than half of those who died were children younger than five years [1], [2]. Of malaria deaths, 92% occurred in sub-Saharan Africa (SSA), where *Plasmodium (P.) falciparum*, the most virulent form of the malaria parasite, is most common. Artemisinin-based combination therapies (ACTs) are recommended by the World Health Organization (WHO) as the first-line treatment for uncomplicated *P. falciparum* malaria in all cases except the first trimester of pregnancy [1]. *P. falciparum* has developed resistance to other treatments, leaving ACTs as the only first-line treatment suitable for use among the general population [3], [4]. Aided by a subsidy through the Affordable Medicines Facility-malaria (AMFm), ACTs became more widely available and affordable in seven pilot SSA countries [5]. Widespread access to ACTs in both the private and the public sectors is crucial for averting death and disability from malaria [6], but also increases the likelihood that *P. falciparum* strains resistant to artemisinin derivatives will emerge in Africa, as has already occurred in Southeast Asia [7], [8].

The high frequency of non-adherence to ACTs in SSA is a primary concern for treatment failure and could also lead to drug resistance, particularly in patients with high parasite burdens [4]. In a systematic review of antimalarial adherence studies, Banek et al. found that reported adherence to artemether-lumefantrine (AL) ranged from 38% to 96% and that adherence to artesunate-amodiaquine (AS+AQ) ranged from 48% to 93% [9]. In two studies which included both public and private facilities, Cohen et al. found that 66% of patients adhered to the prescribed dosing of AL in Uganda [10] and Lemma et al. found that 38% of patients adhered to the treatment regimen for AL in Ethiopia [11].

Mobile technologies are increasingly being incorporated into the health sector both in developed and developing countries. Mobile phone ownership in low-income countries increased from 7.9 phones per 100 inhabitants in 2001 to 78.8 phones per 100 inhabitants in 2011, providing a new and relatively inexpensive platform to reach a large proportion of patients in low-income countries [12]. While few studies have examined the effects of text reminders in developing country settings [13], [14], two studies indicate that text reminders improved patient adherence to HIV/AIDS treatment in Kenya [15], [16] and another study indicates that text reminders improve health worker adherence to treatment guidelines for pediatric malaria [17]. We conducted a randomized trial in northern Ghana to assess the impact of mobile phone-based text message reminders on ACT treatment completion. To our knowledge, this randomized controlled trial is the first attempt to evaluate the impact of text reminders to patients on adherence to malaria treatment [18].

## Methods

### Design Overview

This randomized trial took place within a one hour driving radius of Tamale, Ghana, the capital of Northern Region, between July and November of 2011. and Checklist S1 contain the trial protocol and CONSORT checklist.

### Ethics Statement

The review boards at the Harvard School of Public Health and the Ghana Health Services provided ethics approval. All participants provided written informed consent.

### Setting and Participants

The site of the study was the urban area of Tamale, Ghana, and its peri-urban and rural surroundings (see Figure S1 in file S1 for geographic location). Tamale is the capital of Ghana’s Northern Region and has a population of 540,000 people [19]. Malaria is endemic in the area, with 48.3% of children in the Northern Region testing microscopy-positive for malaria in 2011 [20]. While Ghana as a whole is rapidly developing, the Northern region lags behind the rest of the country on literacy and other indicators [19].

The primary ACTs available in Ghana are AL and AS+AQ. AL regimens generally contain six doses, with the number of pills in each dose varying between one and four. AS+AQ regimens contain six tablets taken in three doses. The first dose of both treatments is taken immediately or with the next meal, and subsequent doses are supposed to be taken in 12-hour intervals for AL and in 24-hour intervals for AS+AQ, so that the full treatment course should be completed within 60 hours of treatment initiation, regardless of patient age.

Ghana is also one of seven pilot countries in SSA where ACTs were subsidized through the AMFm, and thus made available at low prices both in the public and private health sectors beginning in August 2010. The average price per adult ACT dose in Ghana fell from $2.74 to $0.94 in public facilities and from $3.42 to $1.13 in private facilities [5]. Ghana’s National Health Insurance Scheme also fully covers ACTs for insured patients at registered facilities. The National Health Insurance Authority estimates that 25.5% of individuals in the Northern Region were active members in 2010 [21]. It is likely that individuals living in and around Tamale were more likely to be registered than those in more rural areas.

The target population was defined as all individuals acquiring ACTs. Data enumerators compiled a complete listing of vendors distributing ACTs within a 30-minute drive of Tamale. As shown in Table S1 in file S1, enumerators compiled a list of 217 ACT vendors within 30 minutes driving time from the center of Tamale. The most common vendor types were licensed chemical sellers (177), followed by pharmacies (15), private clinics (7) and public hospitals (6). Daily Patient and ACT volumes were highest at private and public facilities as well as pharmacies – the three categories together accounted for about two thirds of the estimated total daily volume.

We continuously recruited participants from 11 high-volume facilities, which primarily included clinics and hospitals, in order to ensure high enrollment volumes to achieve sufficient power. Data enumerators made additional recruitment efforts – generally restricted to only a few days – at 73 smaller vendors, which were selected at random from the complete listing of 217 facilities. Figure S2 in file S1 illustrates the spatial distribution of sampled vendors in the larger Tamale region.

### Vendor Recruitment

Study staff visited all selected vendors a week prior to participant recruitment to seek vendor consent to participate in the study and to establish a plan for vendor communication with data enumerators. Participant registration in the text messaging system and enrollment in the study then took place in two steps. First, consenting vendors distributed flyers advertising “free text message information about malaria” to all individuals acquiring ACTs. Second, vendors notified data enumerators assigned to each shop of the ACT acquisition, and data enumerators initiated study enrollment.

### Participant Recruitment

Participant recruitment for the study took place immediately following acquisition of the malaria treatment and vendor distribution of the flyer. Data enumerators sitting near shop exits approached individuals who acquired anti-malarial treatment when notified of the acquisition by vendors. Data enumerators invited the individuals to participate in a household survey focusing on health and informed them that participating in the household survey would be associated with an interview conducted in their household at a later date; no further information was provided regarding the study’s focus on malaria treatment or adherence in order to avoid influencing participants’ behavior or reporting.

Study eligibility was restricted to individuals 18 or older, to individuals purchasing malaria medicine for themselves or someone in their household, to individuals living within a 30-minute drive of the shop, and to individuals able to provide the mobile number for a personal or shared mobile phone. Enumerators informed study participants who met the inclusion criteria about the household interview to take place within the next few months, and, upon consent, enrolled participants in the study. After the initial consent, surveyors conducted a short interview to collect contact information for eligible participants. Both those who were eligible and those who were ineligible completed a short exit interview designed to capture basic demographic information. Individuals who registered in the mobile phone system based on the flyer but who were not eligible for the study received messages but were not enrolled in the study.

### Participant registration in the text messaging system

Participants who received flyers from vendors and enrolled in the text-messaging program in response to the flyer used their own phones to enroll and did not receive any assistance from data enumerators. Registration in the text messaging system was possible either by a short ring (“flashing”) of the number provided on the flyer or by sending an empty text message to the same number. While flashing is free for the user, phone service providers in Ghana require a non-zero balance to initiate any call, and sending a text message costs between 5 and 10 Ghana pesewas (US$ 0·03-US$ 0·06). Registration in the system was confirmed by a text-message stating, “Thanks for registering for Mobile Health Information.” Patients who called or sent a text to the system while enrolled received an additional message stating, “You are already registered. To stop receiving messages, text STOP. Thanks.” If the system received a “STOP” message, it sent a confirmation (“You will not receive any more messages from Mobile Health”) and automatically discontinued sending any further messages.

### Message Design

To develop the content of messages, we reviewed the literature on SMS reminders and attention. We shared a variety of possible messages with participants of 8 focus groups and 24 individual interviews with adult male and female participants in rural and peri-urban Tamale in March 2011. Participants provided feedback on clarity, appropriateness, and predicted effectiveness of the various message options. Based on the feedback obtained in interviews and focus groups as well as in a small pilot study conducted in Cape Coast in June 2011, the research team opted for a simple reminder message (message A) and an additional encouragement component (message B), highlighting the importance of finishing the drugs when malaria symptoms become weak or absent – one of the primary obstacles to regimen completion reported in preliminary field work. The final message treatments were as follows:

**Message A:** “Please take your MALARIA drugs!”.

**Message A+B:** “Please take your MALARIA drugs! Even if you feel better, you must take all the tablets to kill all the malaria.”

### Randomization of Messages

The text-messaging program was based in the programming language Python version 2.6 and was built on a Django platform (the program is open source and can be found at https://github.com/waveswinger34/pactremind). Participants were sequentially (in order of their enrollment) assigned to treatment and control, with every other enrolled subject assigned to either treatment or control in an alternating manner. The text messaging system then further randomized participants within the treatment condition to receive message A or message A+B with equal probability, based on a pseudo-random number draw.

### Message Rollout

All participants in the treatment group received the assigned reminder messages in 12-hour intervals for three days, for a total of 60 hours, overlapping with the typical treatment protocol for ACTs. Participants in the control group received no message during this period. Given that ACT administration is recommended after meals, evening messages were sent at 7pm, while morning messages were sent at 8am. All participants in the control and in the treatment groups received a generic malaria prevention message about bed nets after 120 hours, which ended the text message activities.

### Outcomes and Follow-up

Data enumerators conducted follow-up interviews approximately 72 hours after participants enrolled in the study. Individuals who could not be reached for in-person follow-up interviews after three attempts were contacted by phone for short phone interviews. All study materials were printed in English, the standard language used for written communication in Ghana. Enumerators interacting with subjects during the baseline and follow-up interviews were not aware of treatment assignment prior to the interview.

Adherence to antimalarial treatment is generally based on self-report and observation of pill packets [9], [14]. The primary outcome variable in this study was self-reported completion of the ACT treatment regimen, with the interviewee reporting whether the patient completed all of their doses. To allow for some delays in treatment initiation, all follow-up interviews were scheduled at least 66 hours after the initial visit; therefore, any unfinished dose found during follow-up visits could be considered as evidence of non-adherence. To address concerns regarding potential recall and social desirability biases associated with self-reports and to validate the robustness of the outcome measure [22], [23] study staff collected information on two additional adherence measures.

First, at the beginning of the interview, and before asking patients any questions regarding the preceding illness episode and its treatment, interviewers took a complete inventory of all drugs stored in the households. If participants were willing to show study staff their inventories, interviewers noted the total variety of drugs available in the household and documented the presence of ACTs. While this was not a direct measure of adherence, we expected the drug inventory to be correlated with adherence, and for patients who did not finish their pills to be more likely to still have ACTs at their home. After the drug inventory, interviewers asked respondents to report their overall adherence and to complete a detailed self-report module with the exact quantity of each ACT dose.

Second, at the end of the treatment module, surveyors asked patients to see the original blister pack; if participants were willing and able to share the blister pack, data enumerators observed the number of remaining pills. We compared the main self-report outcome to both the household drug inventories and to the observation of the blister packs to assess the strength of the self-report outcome. We did not incorporate pill packet observation into the final outcome measure due to potential bias in which participants shared pill packets.

### Statistical Analysis

The study was powered to detect a 10% increase in adherence with power 0.9, assuming an alpha of 0.05 and a control group adherence of 60% based on estimated adherence in a similar study in Uganda [10]. We estimated intent-to-treat (ITT) multiple logistic regression models to estimate the impacts of receiving Message A and Message B relative to the control group, controlling for random patient characteristics that could influence treatment adherence and clustering standard errors by vendor. The covariates included were sex of the patient, patient age, whether the household head was male, educational attainment of the household head, household wealth quintile, and the type of vendor where participants sought treatment. The household wealth index was constructed using principal components analysis based on household cooking arrangements, water source, sanitation, as well as ownership of mobile phones, and air conditioners. Household head educational attainment was classified as no education if the individual had zero years of education, some education if the individual attended primary or secondary school, and higher education if the individual attained undergraduate or other education after secondary school. In addition to the main analysis, we conducted a pre-specified subgroup analysis by age, sex, household head education, wealth, and vendor type using logistic regression. To check the validity of the outcome measure, we tabulated the proportion of patients reporting treatment completion or incompletion against observed pills and observed ACT stock. Finally, we assessed the impact of treatment on still feeling ill during the follow-up interview using logistic regression with covariates. All analysis was conducted using the Stata 11 statistical software package [24].

### Trial Registration

This trial is registered as trial NCT01722734 at clinicaltrials.gov. We regret that we registered the trial late because we were not aware of the requirement to register randomized trials. There are no ongoing trials related to this study, and any future trials related to this study will be registered.

## Results

### Enrollment

**Figure 1:**
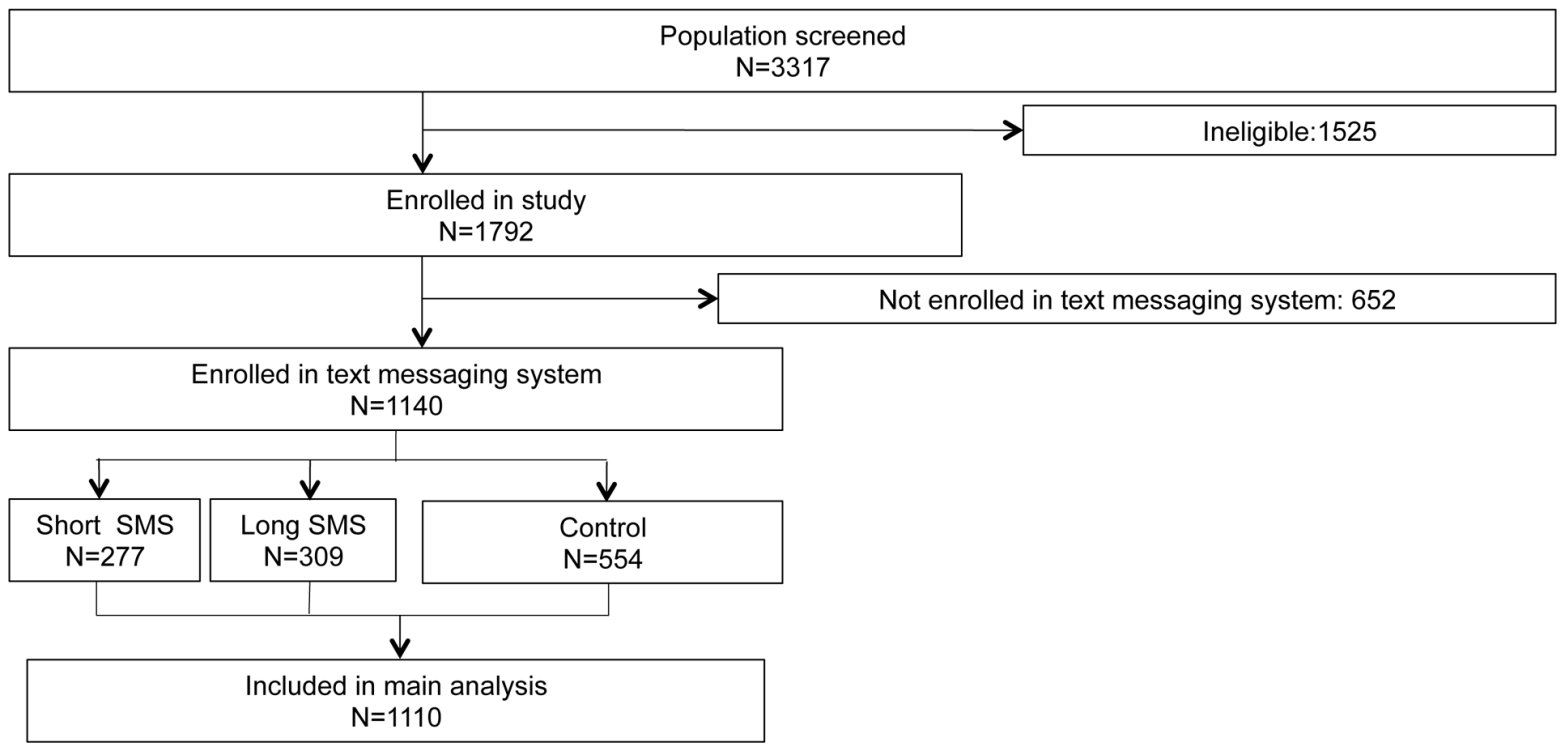
Study Flow. Notes: 1792 of 3317 screened individuals were eligible to participate in the study. The most common reasons for exclusion were living more than a 30-minute drive from the ACT vendor and not having a mobile phone, followed by purchasing drugs for somebody who was not a member of the same household. Of those eligible for study participation, 1140 enrolled with the same mobile phone number that they shared with data enumerators, of whom 554 were randomized to the control group, 277 to the reminder-only message group, and 309 to the reminder and additional information message group. A total of 1110 participants reported on their ACT adherence and were included in the main analysis.

Figure 1 provides an overview of the study. Of 3317 individuals screened for participation, 1525 were ineligible for study participation. The most common reason for exclusion was distance to the facility, with 27% of screened subjects excluded because they lived further than 15 miles from the city of Tamale. Of those who were screened, 11% were excluded because they could not report a mobile phone number at which they could be regularly reached. An additional 18% of those screened were excluded because they bought the drugs for somebody who was not part of their household, because they were under the age of 18, or because they were not willing to participate in the study.

Data enumerators conducted baseline and follow-up interviews with all 1792 eligible participants. Of those eligible, 1140 (63%) enrolled in the SMS system using the same phone number they shared with enumerators. Out of the 652 participants who did not register in the SMS system using the phone number they shared with enumerators, 197 (37%) self-reported successfully enrolling in the system; however, without a matching phone number, we were unable to determine their treatment assignment and did not include them in the analysis. The primary reason subjects reported not being able to register in the mobile system during the follow-up interview was a lack of credit (29%); even though enrollment in the system was designed to be free, “flashing” or texting requires having credit on the phone. The other main reasons for non-enrollment were forgetting the phone at home (23%) and lack of time (14%). Less than ten percent of non-enrollees reported difficulty with the network or phone coverage as the principal reason for not enrolling.

Of the 1140 participants enrolled in the text messaging system using the phone numbers shared with data collectors, 1128 (99.3%) were followed-up in person, 8 (0.7%) were followed up via phone call, and 4 (0.4%) were lost to follow-up. In addition to the 4 lost to follow-up, a further 26 subjects (2.3%) were excluded from the analysis due to missing data on treatment completion, resulting in a total analysis sample of 1110 participants.

Table S1 in file S1 shows enrollment by vendor type. The distribution of the sample population in terms of vendors is fairly similar to the overall patient population of Tamale, with some overrepresentation of patients from public health facilities and underrepresentation of patients from pharmacies.

### Descriptive Statistics

Table 1 shows baseline characteristics of the population enrolled in the trial and the populations excluded from the trial. Individuals were excluded from the trial either due to ineligibility or because they did not register for the text messaging system using the phone number that they shared with data enumerators. The characteristics of the control group and the treatment group are similar, though there were minor differences in age structure, with slightly more subjects of age 5–17 years in the treatment group than in the control group and more subjects of age 18–60 in the control group than in the treatment group. The characteristics of individuals who were eligible for the study but did not enroll in the text message system using the phone number shared with providers are similar to those of individuals who were included in the main analysis. Those excluded from the study due to ineligibility were more likely to be in lower wealth quintiles and had household heads with lower educational attainment than those in the main analysis.

**Table 1:** Sample Characteristics.

	Control		Treatment		Eligible but without mobile match		Ineligible	
	n	% (SD)	n	% (SD)	n	% (SD)	n	% (SD)
Patient is male	213	46.6 (73.9)	225	44.3 (70.5)	301	40.3 (79.7)		
Patient age <5	76	13.7 (35.2)	97	16.5 (36.6)	94	14.8 (32.3)	239	16.6 (36.4)
Patient age 5–17	90	16.3 (36.6)	124	21.1 (41.0)	110	17.3 (36.6)	304	21.1 (40.0)
Patient age 18–59	350	63.4 (48.3)	329	56.1 (49.6)	390	61.4 (49.9)	800	55.5 (50.0)
Patient age 60+	31	5.6 (23.4)	23	3.9 (20.6)	41	6.5 (23.1)	99	6.9 (24.6)
Male household head	498	89.9 (30.2)	511	87.2 (33.4)	571	87.6 (33.0)	1311	85.9 (34.7)
Head of hh: no education	202	39.8 (48.1)	226	42.1 (48.7)	237	40.5 (48.1)	641	48.8 (49.4)
Head of hh: some education	200	39.4 (48.1)	209	38.9 (47.9)	207	35.4 (46.5)	408	31.1 (44.2)
Head of hh: higher education	106	20.9 (39.4)	102	19.0 (37.9)	141	24.1 (41.2)	264	20.1 (37.8)
Household poorest quintile	107	19.3 (39.5)	102	17.5 (37.9)	99	15.2 (35.9)	352	23.7 (34.7)
Household second quintile	100	18.1 (38.5)	94	16.1 (36.7)	133	20.4 (40.3)	324	21.8 (42.1)
Household third quintile	122	22.1 (41.5)	137	23.5 (42.4)	134	20.6 (40.4)	265	17.8 (40.1)
Household fourth quintile	115	20.8 (40.6)	136	23.3 (42.3)	137	20.1 (40.8)	266	17.9 (37.9)
Household wealthiest quintile	109	19.7 (39.8)	114	19.6 (39.6)	146	21.0 (41.7)	280	18.8 (37.9)
Artemether Lumefantrine	317	51.3 (49.5)	326	55.6 (49.7)	379	58.1 (49.4)	782	51.3 (50.0)
Artesunate Amodiaquine	147	26.5 (44.2)	169	28.8 (45.3)	189	29.0 (45.4)	435	28.5 (45.2)
Private hospital	128	23.4 (42.4)	129	22.3 (41.7)	176	27.8 (44.8)	354	23.9 (42.6)
Private clinic	54	9.9 (29.9)	50	8.7 (28.1)	44	6.9 (25.4)	142	9.6 (29.4)
Public hospital	78	14.3 (35.0)	90	15.6 (36.2)	95	15.0 (35.7)	226	15.2 (40.0)
Public clinic	120	21.9 (41.4)	131	22.7 (41.4)	134	21.1 (40.9)	382	25.8 (43.7)
Pharmacy	13	2.4 (15.2)	11	1.9 (13.7)	12	1.9 (13.6)	23	1.6 (12.4)
Licensed Chemical Seller	139	25.4 (4.4)	152	26.3 (44.1)	168	26.5 (44.2)	311	21.0 (40.7)

*Notes:* n is the number of participants in each group who have each characteristic, such as age younger than five years. The denominator for each measure differs based on the number of participants for which an answer was recorded. We did not inquire about patient sex in the screening interview and do not have this information for individuals who were not eligible to participate in the study.

While mobile phone ownership was high, with only 11% of individuals screened not able to share a mobile phone number at which they could be reached, text messaging on phones was not widespread. Only 323 (28.3%) of study participants reported sending any text messages within the last week.

### Adherence

**Figure 2:**
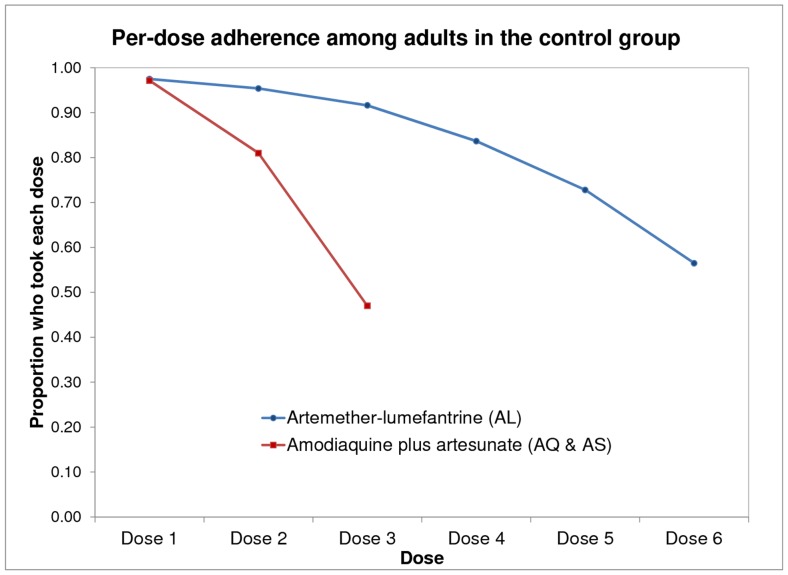
Dose completion among adults in the control group. Notes: Figure 2 indicates the proportion of adults in the control group who reported taking each dose of treatment in the per-dose self-report. Among 239 adults who took AL and reported per-dose adherence, 200 (83.7%) reported taking the fourth dose, 174 (72.8%) reported taking the fifth dose, and 135 (56.5%) reported taking the sixth dose. Among 70 adults in the control group taking AS+AQ, 33 (47.9%) reported completing the full three-dose regimen.

Figure 2 summarizes basic dose completion patterns for adult participants in the control group. Among 239 adults who took AL and reported per-dose adherence, 92.0% reported completing the first three doses. Adherence to the prescribed treatment rapidly declined starting with the fourth dose. Among adults in the control group, 200 (83.7%) reported taking the fourth dose, 174 (72.8%) reported taking the fifth dose, and 135 (56.5%) reported taking the sixth dose. Among 70 adults in the control group taking AS+AQ, 33 (47.9%) reported completing the full, three-dose regimen.

### Message Impact on Adherence

Table 2 shows the main results for the impact of text reminders on self-reported treatment completion. Of 538 participants in the control group, 61.5% report treatment completion. Of 572 participants in the treatment group, 66.4% report treatment completion. According to the ITT analysis, being sent Message A significantly increased the odds of adherence (adjusted OR 1.45, 95% CI [1.03 to 2.04], p-value 0.033). Being in the treatment group to which Message B was also sent did not result in a significant change in the odds of adherence relative to the control group (adjusted OR 0.77, 95% CI [0.50 to 1.20], p-value 0.252).

**Table 2:** Effect of text message treatment on self-reported adherence.

	Percent completed treatment	Adjusted OR(95% CI)
**Control Group** (n = 538)	61.5	Reference Group
**Message A** (n = 572)	66.4	1.45**
		(1.03–2.04)
**Message B** (n = 304)	64.1	0.77
		(0.50–1.20)
**Sample size**	1110	1110

*Notes:* Covariates are all those listed in Table 1, including sex and age of the patient, and household characteristics. **p<0.05.

### Pre-specified Subgroup Analysis

Table 3 shows subgroup analysis by age, sex, household head education, household wealth quintile, type of vendor, and type of drug. Baseline levels of adherence differed, particularly by age, household head education, and vendor type. Adherence was greater among children in the control group (71.4%) than adults in the control group (57.6%), as well as among patients whose household heads have higher education (71.3%) than some education (60.9%) or no education (58.0%). More patients who attended public clinics adhered (69.7%) than those who attended public hospitals (65.3%), private clinics (61.5%), private hospitals (61.1%), or LCSs (58.0%).

**Table 3:** Per-protocol Subgroup Analysis.

	Control	Reminder text message		Long message	
	Proportionadhering (%)	Proportionadhering (%)	Adjusted OR(95% CI)	Proportionadhering (%)	AdjustedOR (95% CI)
**Age**					
** Children**	**71.4**	**76.9**	2.30**	**70.5**	0.44**
(N)	(164)	(215)	(1.01–5.26)	(112)	(0.20–0.99)
** Adults**	**57.3**	**60.7**	**1.26**	**60.9**	**0.96**
(N)	(373)	(352)	(0.92–1.72)	(189)	(0.64–1.42)
**Sex**					
** Male**	**63.1**	**70.5**	**1.35**	**70.7**	**1.05**
(N)	(206)	(220)	(0.86–2.13)	(116)	(0.4–1.74)
** Female**	**57,6**	**66.2**	2.11***	**60.5**	**0.58**
(N)	(238)	(278)	(1.20–3.75)	(147)	(0.28–1.18)
**HH head education**					
** None**	**57.9**	**64.2**	2.02**	**57.8**	0.44**
(N)	(195)	(218)	(1.14–3.56)	(109)	(0.23–0.82)
** Some**	**60.9**	**66.8**	**1.23**	**69.5**	**1.45**
(N)	(197)	(205)	(0.67–2.24)	(105)	(0.63–3.36)
** High**	**71.3**	**72.0**	**1.38**	**66.1**	**0.46**
(N)	(101)	(100)	(0.57–3.37)	(59)	(0.18–1.21)
**Wealth Quintile**					
** Poorest**	**65.7**	**69.3**	**1.02**	**72.0**	**1.35**
(N)	(105)	(101)	(0.37–2.85)	(50)	(0.43–4.24)
** 2^nd^**	**61.2**	**69.9**	2.55**	**66.0**	**0.47**
(N)	(98)	(93)	(1.09–5.96)	(50)	(0.18–1.23)
** 3^rd^**	**58.5**	**64.1**	**1.69**	**65.1**	**0.91**
(N)	(118)	(131)	(0.77–3.69)	(41)	(0.44–1.89)
** 4^th^**	**65.5**	**64.7**	1.77**	**57.7**	0.40***
(N)	(110)	(133)	(1.05–2.98)	(78)	(0.25–0.66)
** Wealthiest**	**57.6**	**66.7**	**1.29**	**65.8**	**0.93**
(N)	(106)	(111)	(0.63–2.63)	(61)	(0.27–3.16)
**Facility**					
** LCS (**N)	**58.0**	**57.1**	**0.79**	**62.3**	**1.51**
	(138)	(147)	(0.44–1.40)	(76)	(0.75–3.04)
** Private**	**61.1**	**75.0**	2.07***	**70.8**	0.73***
** Clinic** (N)	(54)	(48)	(1.47–2.91)	(24)	(0.60–0.88)
** Private**	**61.5**	**67.2**	2.00***	**59.1**	0.38***
** hospital** (N)	(122)	(128)	(1.54–2.60)	(66)	(0.29–0.52)
** Public**	**69.7**	**77.5**	**1.69**	**72.7**	0.36***
** clinic**	(76)	(89)	(0.81–3.53)	(44)	(0.19–0.69)
(N)					
** Public**	**65.3**	**66.7**	**1.39**	**63.8**	**0.73**
** hospital**	(118)	(126)	(0.91–2.13)	(44)	(0.28–1.85)
(N)					
**Treatment**					
** AL**	**0.61**	**0.64**	**1.51**	**0.61**	**0.67**
(N)	(305)	(320)	(0.88–2.59)	(174)	(0.34–1.33)
** AS+AQ**	**0.625**	**0.71**	2.25**	**0.64**	0.41***
(N)	(144)	(163)	(1.17–4.30)	(79)	(0.22–0.78)

*Notes:* N is the number of people in each subgroup, such as the number of children whose caregivers received the simple reminder message. Covariates are all those listed in Table 1, including sex and age of the patient, and household and facility type. There were fewer than 30 individuals in the main analysis who received ACTs from pharmacies, health posts, CHPs zones, or health professionals’ homes. ***p<0.01, **p<0.05.

Our findings indicate that receiving the simple Message A had a significant impact among children, women, individuals with household heads with no education, individuals living in household in the second lowest wealth quintil, individuals obtaining antimalarial treatment from private hospitals, and individuals taking AS+AQ. Receiving Message B significantly increased adherence among individuals obtaining antimalarial treatment from pharmacies and significantly reduced adherence among children, individuals whose household heads had no education, individuals obtaining antimalarial treatment from private hospitals, and individuals taking AS+AQ.

### Reliability of Self-reported Adherence Measures

Study staff collected drug inventories for 929 (83.7%) of 1110 participants included in the analysis. Data enumerators were also able to inspect 667 (60.1%) blister packets. Table 4 shows that data enumerators observed remaining pills for 6.4% of subjects reporting ACT completion and a stock of ACTs for 37.1%, while data enumerators observed remaining pills for 97.9% of participants who did not report completing treatment and a stock of ACTs for 72.1%.

**Table 4:** Self-reported treatment completion and remaining pills or ACT Stock.

	Participant reports treatment completion(N = 711)	Participant reports incomplete treatment(N = 399)
**Pills found in blister back (%)**	6.4	97.9
**ACTs found as part of drug inventory (%)**	37.1	72.1

*Notes:* The proportion of participants who reported adherence and non-adherence and who either had pills remaining in an observed packet or had an ACT stock in the home. Enumerators observed pill packets for 667 (60.1%) participants and drug stocks for 929 (83.7% of) participants.

### Message impact on patient health

Table 5 shows the associations between treatment and the patient's health status as reported during the follow-up interview. 339 respondents (30.2%) reported that the patient still experienced malaria or fever symptoms when interviewed. Receiving a text reminder did not have a significant impact on the odds of remaining sick during the follow-up interview (adjusted OR 1.06, 95% CI [0.78–1.45], p-value 0.700).

**Table 5:** Effect of treatment on still feeling sick.

	Proportion completed treatment%	Adjusted OR(95% CI)
**Control Group (**n = 538)	29.5	Reference group
**Message A (**n = 577)	30.9	1.06
		(0.78–1.45)
**Message B (**n = 303)	30.7	1.02
		(0.79–1.30)
**Sample size**	1110	1110

*Notes:* Patients reported still feeling sick at the time of the follow-up interview. Logistic regression includes the full set of covariates listed in Table 1, including sex and age of the patient, household and facility characteristics.

## Discussion

The results presented in this paper have two main implications. First, adherence to ACT treatment regimens is low in Ghana, which is consistent with other studies of antimalarial treatment adherence in SSA [9]–[11]. On average, only 61.0% of patients in the control group reported completing the full regimen of AL, and 62.5% reported completing the full regiment of AS+AQ. The low overall rate of adherence poses a threat to patient health and to the sustainability of current anti-malaria efforts.

Second, the results presented in this paper suggest that receiving a simple reminder stating, “Please take your MALARIA drugs!” significantly increases adherence to antimalarial treatment. The additional message stating, “Even if you feel better, you must take all the tablets to kill all the malaria” did not have a statistically significant impact on adherence. The finding that our message with more content was not more effective than our shorter message is consistent with the results in Pop-Eleches et al. [16]. The power to detect additional message impacts was, however, limited in our setting; further research is necessary to better understand the most effective content of text message reminders for increasing medication adherence.

Third, we find that different message content can play an important role among subgroups. The simple reminder message appeared to be particularly effective in increasing treatment completion among children, women, individuals whose household heads had no education, individuals in the second lowest wealth quintile, individuals acquiring ACTs from private hospitals, and individuals taking AS+AQ. Receiving the additional message about taking all of the tablets significantly reduced treatment completion among children whose parents or caregivers received the message, individuals whose household heads had no education, individuals obtaining antimalarial treatment from private hospitals, and individuals taking AS+AQ. Message A was significantly more effective in increasing treatment completion than Messages A+B for children whose caregivers received the messages, for individuals whose household heads had no education, for individuals acquiring ACTs from private hospitals, and for individuals taking AS+AQ. These results suggest that it is important to understand the mechanisms driving the impact of text message reminders on different subgroups and to carefully design the content of text message reminders based on target populations.

Receiving a text message did not have an impact on the odds of still feeling sick during the follow-up interview. Neither the short message nor the long message impacted still feeling sick during the follow-up interview. This is consistent with the magnitude of the impact of the messages on adherence and the magnitude of the impact of adherence on still feeling sick. The objective of this intervention is not only to improve individual symptoms, but to encourage behaviors beneficial for population health in the long run – in this case, by decreasing the likelihood that artemisinin-resistant parasites may survive and be spread due to patients ending their treatment too early [4].

### Strengths and Limitations

One of the main critiques of mobile health programs is that they can be difficult to scale. Our results indicate 63.6% of eligible participants self-enrolled in the mobile health program and that flyers are a feasible method of recruiting participants for a mobile health intervention. Enrolling participants in the text message system through a flyer also implies that individuals not able or unwilling to self-enroll in the mobile information system were excluded from the study and from benefiting from the intervention. In terms of the study population, the self-selected nature of patients in the study means that the results presented do not represent treatment effects for the average (or a representative) population, but rather represent the potential SMS impact on adherence among subjects who would self-enroll in a reminder system.

We also could not match individuals to their treatment unless the phone number they provided matched a phone number in the system. The study would have been strengthened by asking participants if they had multiple phone numbers and through additional methods of tracking randomization assignment.

One of the limitations of this study, and text message programs in similar settings more generally, is the importance of mobile phone literacy. While only 11% of households had to be excluded from the study because of lack of access to phones, only 28.3% of participants had sent a text message within the past week. The effectiveness of the program may have been limited by mobile phone literacy.

The intervention, by nature of its delivery via personal mobile phones, is limited in its ability to reach those of lowest socioeconomic status. Mobile phone ownership among people of low socioeconomic status may increase as mobile service and mobile phones become increasingly ubiquitous. There is promising evidence that the short text message had a greater impact on adherence among patients whose household heads had no education, as well as among females and children. There was, however, no clear trend across wealth quintiles.

It is also worth highlighting that similar interventions may yield different results in other developing country settings. Compared to other developing countries, Ghana has made fast progress both with respect to income and average education levels of its population; nevertheless, education and literacy levels remain low among the adult population, particularly in northern Ghana. Better results seem feasible in settings with higher levels of literacy and more frequent mobile phone use.

Finally, the study is somewhat limited by its use of self-reported adherence as a primary outcome measure. Even though all evidence collected as part of this study suggests that systematic misreporting is likely limited and balanced across treatment groups, self-report does present a risk of inaccuracies due to bias and imperfect memory.

## Conclusions

The results of this study suggest that receiving a simple text message reminder can increase adherence to antimalarial treatment. Further research is needed to develop the most effective text message reminder content and frequency.

## Supporting Information

File S1 **Supporting files. Figure S1,** Location of Study Site. **Figure S2,** Each star represents one vendor. Darker stars represent higher patient volumes. **Table S1,** Vendor and Sample Volumes.
(DOCX)

Checklist S1 **CONSORT checklist.**
(DOC)

Protocol S1 **Trial protocol.**
(DOCX)
