# Supplementary material for: The Impact of Text Message Reminders on Adherence to Antimalarial Treatment in Northern Ghana: A Randomized Trial
Source: PLoS One. 2014 Oct 28;9(10):e109032. doi: 10.1371/journal.pone.0109032 (PMC4211682; doi:10.1371/journal.pone.0109032)
Supplement: File S1 — Supporting files. Figure S1, Location of Study Site. Figure S2, Each star represents one vendor. Darker stars represent higher patient volumes. Table S1, Vendor and Sample Volumes. (DOCX) [file pone.0109032.s001.docx]

**Figure S1: Location of Study Site**

**
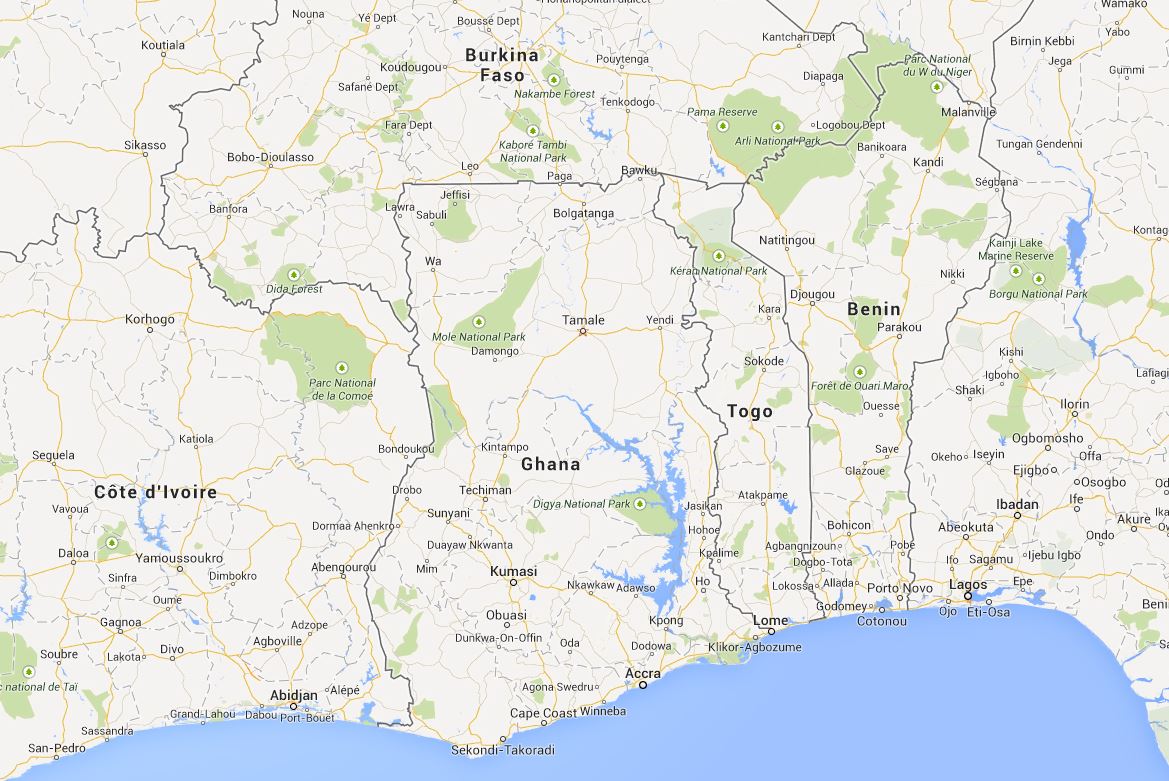
**

**Figure S2: Spatial Distribution of Vendors**

**
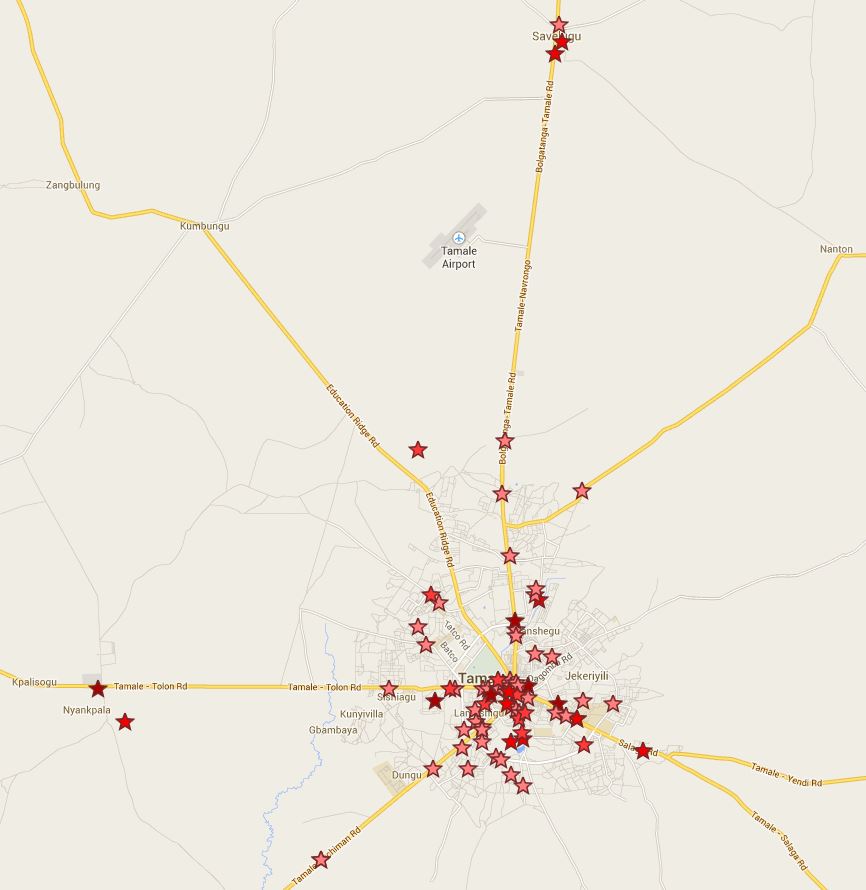
**

**Table S1: Vendor and Sample Volumes**

| Vendor type | Median number of patients per day^a)^ | Number of facilities listed | Total daily volume | Share of patient volume | Sample Share  (N=1140) | Difference |
| --- | --- | --- | --- | --- | --- | --- |
| Pharmacy | 48.3 | 15 | 724 | 29.7% | 12.3% | -17.4% |
| Drug shops | 3.6 | 177 | 637 | 26.1% | 27.4% | 1.2% |
| Private hospital | 69.3 | 7 | 485 | 19.9% | 22.5% | 2.6% |
| Public clinic | 58.0 | 2 | 116 | 4.8% | 13.2% | 8.4% |
| Public hospital | 73.6 | 6 | 442 | 18.1% | 22.0% | 3.9% |
| Health posts | 5.7 | 4 | 23 | 0.9% | 0.8% | -0.1% |
| Health worker home | 10.5 | 1 | 11 | 0.4% | 1.8% | 1.4% |

1. Median nu*m*ber represents median number among shops sampled in the study.
