# Supplementary material for: The Impact of Text Message Reminders on Adherence to Antimalarial Treatment in Northern Ghana: A Randomized Trial
Source: PLoS One. 2014 Oct 28;9(10):e109032. doi: 10.1371/journal.pone.0109032 (PMC4211682; doi:10.1371/journal.pone.0109032)
Supplement: Protocol S1 — Trial protocol. (DOCX) [file pone.0109032.s003.docx]

**Preserving artemisinin-based Combination Therapies (ACTs) Study Protocol**

**Principal Investigators:**

Julia Goldberg, Harvard School of Public Health

[JuliaRGoldberg@gmail.com](mailto:JuliaRGoldberg@gmail.com)

Günther Fink, Harvard School of Public Health

[gfink@hsph.harvard.edu](mailto:gfink@hsph.harvard.edu)

**Study Staff**

Heather Lanthorn, research manager

Slawa Rokicki, intern

**Implementing organizations:**

Harvard School of Public Health

Innovations for Poverty Action ‐ Ghana

**Funding:**

Clinton Health Access Initiative

The funders of the study did not play a role in designing the study protocol, in collecting data, in analyzing data, in interpreting data, or in writing the report. Both authors have full access to all the data from the study and conducted the analyses independently.

**ABSTRACT**

Artemisinins are currently the only effective malaria medication suitable for widespread distribution.[^1^](#_ENREF_1) Artemisinin-based combination therapies (ACTs) combine artemisinins with other treatments, so that the other drug should kill any parasites not killed by the artemisinin. Completion of the full ACT treatment regimen is critical to preventing parasite resistance.

We investigate whether sending text-message reminders to patients’ mobile phones could represent a simple and low‐cost method of increasing adherence to antimalarial regimens through a randomized controlled trial in Tamale, Ghana. This technique could be particularly useful in sub-Saharan Africa (SSA), the region with the highest rates of malaria morbidity and mortality – and with the fastest growing rate of mobile phone users.

**BACKGROUND**

**Preventing resistance to antimalarial treatment**

Malaria killed at least 800,000 people in 2009, 90% of whom lived in Africa [1]. The *Plasmodium falciparum* parasite, responsible for the majority of malaria mortality, has developed widespread resistance to all classes of antimalarial drugs suitable for widespread use except for artemisinins[2]. In some places, researchers have already seen resistance to artemisinin monotherapy [3,4]. In the past, declines in child survival have followed the emergence of widespread resistance to antimalarial treatments [5]. *P. falciparum* develops resistance when a random genetic mutation renders a parasite less susceptible to a particular antimalarial. To lessen the likelihood that *P. falciparum* develops resistance, artemisinin-based combination therapies (ACTs) partner artemisinin with other antimalarial treatments.[^6^](#_ENREF_6) The partner drug should kill any parasites not wiped out by the artemisinin, but patients must adhere to the full treatment regimen for the partner drug to be effective [6]. In this randomized trial, we study the impact of text message reminders on ACT regimen adherence in Tamale, Ghana.

Few researchers have found reliable means of improving adherence, a widespread problem with all varieties of medication [7]. Medication adherence in general is thought to be low, with an estimated 50% of patients adhering to treatment regimens [8]. While the reasons for non-adherence are likely to vary across settings, forgetting appears to be one of the primary causes; in a study of hypertension patients in the United States, 79% of non-adherent patients cited forgetting as their main reason for not adhering to hypertension treatment [9]. Even though the typical ACT regimen of two doses a day for three days is relatively short compared to other medication regimens, there is evidence that patients do not adhere to the ACT regimen. Cohen and colleagues found that only 66% of malaria patients in Uganda were probably adherent to the artemether-lumefantrine (AL) form of ACTs [10].

There are ongoing efforts to prevent *P. falciparum* from developing resistance to ACTs. The Affordable Medicines for malaria (AMFm) initiative funds subsidized ACTs, counteracting consumption of less effective treatments or of counterfeit ACTs which may cost less, but contain artemisinin-based monotherapies or suboptimal levels of medications. In Uganda, researchers from the Harvard School of Public are also studying manners of increasing adherence to ACT regimens through treatment packaging.

Text message reminders complement other efforts to improve adherence and provide an opportunity to both cue patients to increase their attention to treatment and to provide information on treatment regimens and the importance of adherence. In reviews of interventions to improve treatment adherence, Haynes and colleagues and McDonald and colleagues found that educational interventions improved adherence [7,11]. Karlan and colleagues found that text message reminders that brought participant attention to monetary savings increased savings [12]. The increasing ubiquity of mobile phones provides unprecedented opportunities for researchers to examine new ways of delivering health services to people, particularly among disadvantaged populations. Mobile phone subscriptions per 100 people increased from an estimated 0.68 in the year 2000 to 71.49 in 2010 [13].

In this study, we seek to evaluate the impact of text-message reminders as a scientifically promising, inexpensive, and increasingly accessible approach to improving ACT adherence.

**Objectives**

The primary objective of the study is to assess the impact of text message reminders on adherence to ACT regimens of two doses a day for three days. The secondary objectives are to develop a scalable method of delivering text message reminders for short-term treatment and to better understand the mechanisms that drive adherence and non-adherence to antimalarial treatment.

**Overview of Study Design**

This single-blinded randomized controlled trial takes place in Tamale, Ghana. Malaria patients can acquire ACTs without a prescription from a range of public and private health providers in Ghana, a system that is similar to many countries where there is a high incidence of malaria. Participating vendors will distribute flyers advertising free enrollment in a mobile health information system to anyone who purchases antimalarial treatment. Having patients enroll themselves in the text message reminder system, on their own phones, and based on a flyer is meant to represent a method of enrollment that would be scalable if the intervention is successful. Participants who self-enroll in the text messaging system will be randomized at the level of the individual and will be automatically assigned by the phone system to the treatment group or to the control group with equal probability. Participants randomized to the treatment group will receive one reminder for each of the 6 doses of malaria treatment, and will be further randomized with equal probability to receive a short message or a long message. To assess the impact of the program on adherence, study staff will enroll patients into the study upon patient exit from ACT vendors. Study staff will visit participants three days after study enrollment to assess adherence, health outcomes, and basic sociodemographic information.

**STUDY DESIGN**

**Study Setting**

The site of the study is the area around the city of Tamale, Ghana. Tamale is a city of 540,000 people [14]. The area around Tamale quickly transitions into a rural environment, so that urban, peri-urban, and rural populations will be included in the study. Ghana is an AMFm pilot country and the ACTs artemether lumefantrine (AL) and artesunate amodiaquine (AS+AQ) are widely available and highly subsidized. ACTs are also covered by Ghana’s National Health Insurance Scheme, so that many people do not have to pay for ACTs. Malaria is a leading cause of morbidity and mortality in Ghana, particularly for young children. Malaria incidence usually peaks in Ghana’s rainy season of July and August, when we will begin the study.

**Target Population**

The target population for this study is defined as all individuals who reside within a thirty minute drive of Tamale, Ghana, who obtain ACTs for the treatment of malaria in their household from either public health facilities or through the retail sector.

**Study Recruitment Location**

Data enumerators will recruit study participants at hospitals, clinics, pharmacies, and licensed chemical seller (LCS) in Tamale, Ghana. All vendors of ACTs will be listed through an initial census. Subjects will be recruited from randomly selected vendors as described in further detail in the Study Enrollment section.

**Eligibility Criteria**

Participants must meet the following eligibility criteria to participate in the study:

1. Individuals must have access to their own or to a shared phone and be able to provide the phone number.
2. Individuals must reside within a thirty minute drive of the vendor from which they receive ACTs.
3. Individuals must be age 18 years or older
4. Individuals must be purchasing ACTs

**Study Enrollment**

At the beginning of each day of recruitment, trained study staff will visit selected vendors, who will be informed about a new cell phone based program which will provide free information on malaria to their customers. Study staff will provide vendors with a stack of flyers encouraging people purchasing malaria medicine to enroll into a text message program, which will provide “text message information on malaria,” as illustrated in Figure 1.

**Figure 1: Flyer advertising text message information system**

Vendors will signal study staff if any patient purchases antimalarial treatment. Study staff will then approach all these individuals, assess study eligibility, and collect basic demographic information. Study staff will ask individuals eligible to participate in the study for informed consent to participate in the full study and to visit their homes at some point in the next three months. Those who consent to participate in the larger study will be asked for contact information, including mobile phone number, and for directions to their home.

**Text Reminder System Registration**

Enrollment in the text message system is free of charge. Enrollment in the text message system can be accomplished both by flashing (calling and letting it ring once or twice) or texting in to the system number, as provided on the flyer (Figure 1).

Upon registration, all enrollees will receive a “thank you for registering” message promptly after submitting a phone number into the system. This message will also provide the recipient with information on how to stop receiving messages at any time (by texting “STOP” to the system phone number).

**Text Message Treatments**

After registration, the automated text message system (programmed in Java) will randomly assign individuals to the three treatment groups. Participants in the treatment groups will receive a total of six messages, at 07:00 and 19:00 each day, consistent with the ACT treatment regimen of two doses a day for three days. The comparison group will receive no messages apart from the initial enrollment message and a final generic message about malaria prevention. The first malaria message will be delayed to 15 minutes after enrollment if it occurs prior to 15:00. Respondents enrolling after 15:00 will receive their first message at 19:00 the same day. Within the treatment group, recipients will be randomized to receive a short message or a long message.

The short message will convey a reminder to take the malaria tablets. The long message will be a combination of the short message plus an additional message, which will elaborate that taking the tablets is important even if symptoms have subsided into order to be fully cured. The short message is intended to serve as a simple reminder. The long message is intended to both serve as a reminder and to convey information about the treatment regimen and the importance of completing treatment.

All enrollees will receive a general message about malaria prevention at approximately 120 hours after their initial enrollment.

**Figure 2: Message Flow**

**Blinding**

The study is single-blinded, with interviewers blinded to treatment prior to end of the follow-up interview, when participants are directly asked about whether they received text messages and, if they received messages, their impressions of the text messages. The participants cannot be blinded to treatment due to the nature of the treatment, though participants will not know what the different treatments are or the differences between the text messages they receive and what others receive.

**Follow-up Survey and Adherence Assessment**

Surveyors will make home visits to enrolled participants between 72 and 96 hours after the in-vendor recruitment to assess dose-completion and to inquire about patient malarial symptoms, care-seeking patterns, awareness of malaria and AMFm, and other areas related to memory and potential mechanisms. For those randomized to receive text messages, study personnel will inquire about the helpfulness and receptivity to the text messages and their content. For patients who are not located at their homes after three attempts, interviewers will conduct an abbreviated version of the Follow-up Questionnaire over the phone. An auditor will call several days after each follow-up interview to ensure that the interview went smoothly and to cross check one or two questions.

**Outcome measure**

The primary outcome variable for the study is adherence to the ACT treatment regimen.

Given that there are currently no blood tests or other biomarkers available to assess the number of doses taken, establishing adherence to malaria treatment is a non-trivial challenge [7,11]. We will rely primarily on self-reports because direct observation is not feasible. To address concerns regarding likely recall and social desirability biases, we developed a detailed interview protocol, allowing a three-way triangulation of the actual program impact. First, at the very beginning of the interview, and before asking patients any questions regarding the preceding illness episode and its treatment, interviewers will ask to see all drugs stored in the households of consenting participants and will note the presence of ACTs. Later in the follow-up interview, interviewers will guide patients through a detailed medication module, asking participants to provide the exact timing and quantity of tablets in each dose. At the end of the treatment module, surveyors will ask patients for the original blister pack, and, when available, note the number of pills available.

Blister packs will only be available for patients who do not finish their treatment or who hold on to an empty pack. It is also possible that patients who are not honest in the self-reported measure may not be honest about having their pill packet. We therefore will not use observed pill count as the main outcome measure. The primary purpose of collecting the blister pack information is to validate self-reports among the subgroup where blister packs were available. The purpose of the initial drug inventory is also to address the concern of systematic misreporting. Given that study participants will be not aware of any link between the household survey and text message system, it seems unlikely that patients would feel compelled to lie about the presence of medicines in their household, so that unfinished ACTs should be detected in the household inventory. Given that households may have had ACTs in stock from previous treatments, or may have stocked ACTs for future treatment, the ACT stocking measure is not directly a measure of adherence. However, if the program does affect adherence, it should also affect the fraction of households storing unfinished packages.

**Power Calculations**

The study is powered to detect a 10% increase in adherence (from 0.60 to 0.66) with power 0.9 and assuming an alpha of 0.05.

**Ethical considerations**

The Ghana Health Service and the Harvard School of Public Health institutional review boards approved this study and its instruments. Since there is little stigma associated with malaria, the most important ethical consideration in this study is the potential disclosure of identifiers linked to demographic information. Identifiers will be stored in separate files from demographic information to address this issue. Data will be collected on coded surveys by study personnel and stored in a locked file cabinet.

**Data analysis**

De-identified data will be analyzed in STATA [15]. In addition to examining the impact of the short and the long text message reminders on ACT adherence, the analysis will include an examination of different patterns of adherence and of intervention impact based on age and income. Qualitative information will also be analyzed to assess the receptivity to text message reminders for malarial and other medications and the barriers to compliance with treatment.

**REFERENCE LIST**

1. World Health Organization (2010) World malaria report. Geneva, Switzerland: World Health Organization.

2. AMFm Taskforce of the Roll Back Malaria Partnership (2007) AMFm Technical Design.

3. Jambou R, Legrand E, Niang M, et al. (2005) Resistance of Plasmodium falciparum field isolates to in-vitro artemether and point mutations of the SERCA-type PfATPase6. *Lancet* **366**(9501): 1960-3.

4. Phyo AP, Nkhoma S, Stepniewska K, et al. (2012) Emergence of artemisinin-resistant malaria on the western border of Thailand: a longitudinal study. *Lancet* **379**(9830): 1960-6.

5. Cohen JM, Smith DL, Cotter C, et al. (2012) Malaria resurgence: a systematic review and assessment of its causes. *Malaria journal* **11**: 122.

6. White NJ (2004) Antimalarial drug resistance. *The Journal of clinical investigation*; **113**(8): 1084-92.

7. Haynes RB, Ackloo E, Sahota N, McDonald HP, Yao X (2008) Interventions for enhancing medication adherence. *Cochrane Database Syst Rev*; (2): CD000011.

8. Sabaté E (2003) Adherence to long-term therapies : evidence for action. Geneva: World Health Organization.

9. Siegel D, Lopez J, Meier J (2007) Antihypertensive medication adherence in the Department of Veterans Affairs. *The American journal of medicine*; **120**(1): 26-32.

10. Cohen JL, Yavuz E, Morris A, Arkedis J, Sabot O (2012) Do patients adhere to over-the-counter artemisinin combination therapy for malaria? evidence from an intervention study in Uganda. *Malaria journal*; **11**: 83.

11. McDonald HP, Garg AX, Haynes RB (2002) Interventions to enhance patient adherence to medication prescriptions: scientific review. *JAMA : the journal of the American Medical Association*; **288**(22): 2868-79.

12. Karlan DS, McConnell M, Mullainathan S, Zinman J. (2010) National Bureau of Economic Research. Getting to the top of mind how reminders increase saving. NBER working paper series working paper 16205. Cambridge, MA: National Bureau of Economic Research.

13. International Telecommunications Union (2012) Mobile cellular subscriptions per 100 inhabitants, 2001 - 2011. <http://www.itu.int/ITU-D/ict/statistics/material/excel/20112/ictwebsite/Mobile_cellular_01-11.xls>

14. Ghana Statistical Service (2012) 2010 Population and Housing Census. Accra, Ghana.

15. StataCorp. (2009) Stata Statistical Software: Release 11. College Station, TX: LP S.
